# Chromatographic analysis and monitoring of fault gases in substation using ZIF-8 coated capillary column

**DOI:** 10.3389/fchem.2025.1618268

**Published:** 2025-08-07

**Authors:** Hua Mao, Yan Wang, Jie Wang, Zeyao Ma, Yalong Xia, Xiaoliang Zeng, Shihong Hu, Xinsheng Lan, Fangqiang Wang, Lin Zhou, Xuewen Liu

**Affiliations:** ^1^ State Grid Sichuan Electric Power Research Institute, Chengdu, Sichuan, China; ^2^ Power System Security and Operation Key Laboratory of Sichuan, Chengdu, Sichuan, China; ^3^ State Grid Sichuan Electric Power Company, Chengdu, Sichuan, China; ^4^Gaoxin District Branch, Sichuan Shuneng Electric Power Co., Ltd., Chengdu, Sichuan, China

**Keywords:** ZIF-8, capillary column, insulating oil, fault gases, gas chromatography

## Abstract

This study developed a ZIF-8-coated capillary gas chromatography column for the efficient separation of critical fault gases in electrical substations. By optimizing the carrier gas flow rate (3.5 mL/min) and column temperature (40°C), baseline separation of all components was achieved within 12 min, with resolution values of 0.8 and 3.8 for C_3_H_6_/C_3_H_8_ and C_2_H_4_/C_2_H_6_, respectively. The 3.4 Å molecular sieve pores of ZIF-8 preferentially retained larger hydrocarbons (e.g., C_4_H_10_) via size-exclusion effects, while its hydrophobic surface minimized non-specific adsorption of polar molecules like CO_2_. The low polarity of methylimidazole ligands ensured selective retention of nonpolar and weak polar gases. Remarkable reproducibility was demonstrated by retention time relative standard deviations (RSD) < 0.08% across 11 consecutive injections, confirming the coating’s mechanical stability. Coupled with high-temperature resistance and rapid analysis capabilities, this column provides a reliable tool for real-time substation gas monitoring, enabling early warning of faults (e.g., arcing, overheating) and intelligent diagnostics of insulation degradation.

## 1 Introduction

The analysis of gas components in electrical substations is critical for ensuring operational safety and equipment reliability (Gómez-Sandoval et al., 2021). During routine operations or under fault conditions (e.g., partial discharge, overheating), substation equipment such as transformers and circuit breakers may generate characteristic gas mixtures, including methane (CH_4_), ethane (C_2_H_6_), ethylene (C_2_H_4_), propane (C_3_H_8_), propylene (C_3_H_6_), butane (C_4_H_10_), carbon monoxide (CO) (Gao et al., 2024). Monitoring these gases provides early warnings of insulation degradation, arcing, or thermal faults, enabling preventive maintenance and minimizing catastrophic failures (Božiček et al., 2022). For instance, elevated levels of hydrocarbons (e.g., CH_4_, C_2_H_4_) often indicate oil decomposition in transformers, while CO may signal cellulose insulation breakdown. Accurate identification and quantification of these gases are therefore essential for both safety and economic efficiency in power systems (Huaan et al., 2020; Stanković et al., 2022).

Conventional gas chromatography (GC) methods for substation gas analysis typically employ stationary phases such as molecular sieves (Jia et al., 2014), alumina, or porous polymer beads (Liao et al., 2022; Wei et al., 2023; Ouyang et al., 2024). While these materials are widely used, they exhibit notable limitations. Molecular sieves, though effective for separating permanent gases (e.g., H_2_, N_2_), struggle to resolve hydrocarbons and CO within a single analytical run, necessitating column switching or multiple detectors. Alumina columns, while suitable for light hydrocarbons, suffer from poor reproducibility due to moisture sensitivity and limited thermal stability (Han et al., 2024). Porous polymer phases, such as divinylbenzene-based materials, offer broader applicability but often lack the selectivity required to distinguish structurally similar compounds (e.g., C_2_H_4_ vs. C_2_H_6_) or separate low-concentration analytes in complex mixtures (Ahmadi and Kim, 2023). Furthermore, many commercial columns degrade at elevated temperatures, reducing their lifespan and reliability in continuous monitoring applications (Mametov et al., 2021). These challenges underscore the need for advanced stationary phases with enhanced separation efficiency, thermal robustness, and versatility.

Metal-organic frameworks (MOFs), a class of crystalline porous materials constructed from metal ions and organic linkers, have emerged as promising candidates for chromatographic separations due to their tunable pore structures, high surface areas, and chemical stability (Jia et al., 2013; Long et al., 2013; Jia et al., 2015; Jia et al., 2017; Jia et al., 2021; Liu et al., 2021; Gao et al., 2022; Tong et al., 2024). Among MOFs, zeolitic imidazolate frameworks (ZIFs) are particularly notable for their zeolite-like topologies and exceptional stability under harsh conditions (Assfour et al., 2010; Liu et al., 2013; Wang et al., 2013). ZIF-8, composed of zinc ions bridged by 2-methylimidazole ligands, features a sodalite-type structure with a pore aperture of 3.4 Å and a large cavity (11.6 Å), enabling selective adsorption based on molecular size and polarity (Liu et al., 2013). Its hydrophobic nature and resistance to moisture further enhance its suitability for gas-phase applications. Recent studies have demonstrated ZIF-8’s efficacy in separating small molecules, including CO_2_/CH_4_ mixtures and light hydrocarbons, leveraging its precise pore geometry and strong host-guest interactions (Yu et al., 2021). These attributes position ZIF-8 as an ideal stationary phase for substation gas analysis, where simultaneous separation of polar (e.g., CO) and nonpolar (e.g., C_1_–C_4_ hydrocarbons) species is required.

In this study, we report the fabrication of a ZIF-8-coated capillary column for the gas chromatographic analysis of substation gases. The *in situ* synthesis of ZIF-8 on the capillary inner wall ensures a uniform and stable stationary phase, addressing the limitations of conventional dynamic coating methods. The microporous structure of ZIF-8 facilitates size- and polarity-based discrimination, enabling baseline separation of CH_4_, C_2_H_6_, C_2_H_4_, C_3_H_8_, C_3_H_6_, C_4_H_10_, CO, and N_2_ in a single chromatographic run. The column’s performance was systematically evaluated under varying temperatures and flow rates, demonstrating high resolution, reproducibility, and thermal stability up to 300°C. This work highlights the potential of ZIF-8 as a next-generation stationary phase for substation gas monitoring, offering improved analytical efficiency and reliability over existing technologies.

## 2 Experimental

### 2.1 Chemicals and instruments

All reagents were of analytical grade unless otherwise specified. Zinc nitrate hexahydrate (Zn(NO_3_)_2_·6H_2_O, 99%) and 2-methylimidazole (Hmim, 99%) were purchased from Sigma-Aldrich (Shanghai, China). Methanol (HPLC grade), sodium hydroxide (NaOH), and hydrochloric acid (HCl) were obtained from Chengdu Kelong Chemical Company (Chengdu, China). A standard gas mixture containing methane (CH_4_), ethane (C_2_H_6_), ethylene (C_2_H_4_), propane (C_3_H_8_), propylene (C_3_H_6_), butane (C_4_H_10_), carbon monoxide (CO) in nitrogen (N_2_) was supplied by Zhongce Standard Material Co., Ltd. (China). Fused-silica capillaries (0.25 mm i.d., 10 m length) were purchased from Yongnian Optic Fiber Factory (Hebei, China).

Chromatographic separations were performed on a Fuli GC-9720plus Gas Chromatograph (Fuli Instruments Co., China) equipped with a thermos conductive detector (TCD) and a capillary injection port. Helium (99.999%) was used as the carrier gas. Scanning electron microscopy (SEM) images were acquired using a JEOL JSM-7800F microscope (Japan) at 30 kV. Thermogravimetric analysis (TGA) was conducted on a Netzsch STA 449F3 instrument (Germany) under a nitrogen atmosphere (heating rate: 10°C/min).

### 2.2 Synthesis of ZIF-8

ZIF-8 crystals were synthesized via a solvothermal method. Briefly, 2.93 g of Zn(NO_3_)_2_·6H_2_O and 3.28 g of 2-methylimidazole were dissolved separately in 50 mL of methanol. The two solutions were mixed under vigorous stirring for 30 min, yielding a milky suspension. The mixture was then transferred to a Teflon-lined autoclave and heated at 120°C for 24 h. The resulting white precipitate was centrifuged, washed three times with methanol, and dried under vacuum at 80°C for 12 h.

### 2.3 Pretreatment of capillary

To enhance ZIF-8 adhesion, the inner wall of the fused-silica capillary was functionalized with hydroxyl groups. The capillary was sequentially flushed with 1 M NaOH (20 μL/min, 2 h), ultrapure water (until neutral pH), 0.1 M HCl (1 h), and dried under nitrogen flow at 100°C for 12 h. A 1% (v/v) solution of (3-aminopropyl)trimethoxysilane (APTMS) in toluene was then introduced into the capillary at 10 μL/min, sealed, and heated at 110°C for 12 h. The silanized capillary was rinsed with methanol and dried under nitrogen to remove residual reagents.

### 2.4 *In-situ* preparation of ZIF-8-coated column

The ZIF-8 stationary phase was coated onto the capillary via an *in situ* growth method. A precursor solution containing 0.1 M Zn(NO_3_)_2_ and 0.4 M 2-methylimidazole in methanol was prepared and sonicated for 15 min. The solution was infused into the pretreated capillary at 5 μL/min, sealed, and heated at 80°C for 48 h. After synthesis, the capillary was rinsed with methanol to remove unreacted precursors and dried under nitrogen flow.

### 2.5 Column performance testing

The chromatographic efficiency of the ZIF-8-coated column was evaluated using n-dodecane as a test analyte. The Van Deemter curve was generated by measuring the theoretical plate number (N) at carrier gas flow rates ranging from 5 to 45 cm/s. The McReynolds constants were calculated using benzene, n-butanol, 2-pentanone, 1-nitropropane, and pyridine as probe molecules.

## 3 Results and discussion

### 3.1 Characterization of the synthesized ZIF-8 and coated column

**FIGURE 1:**
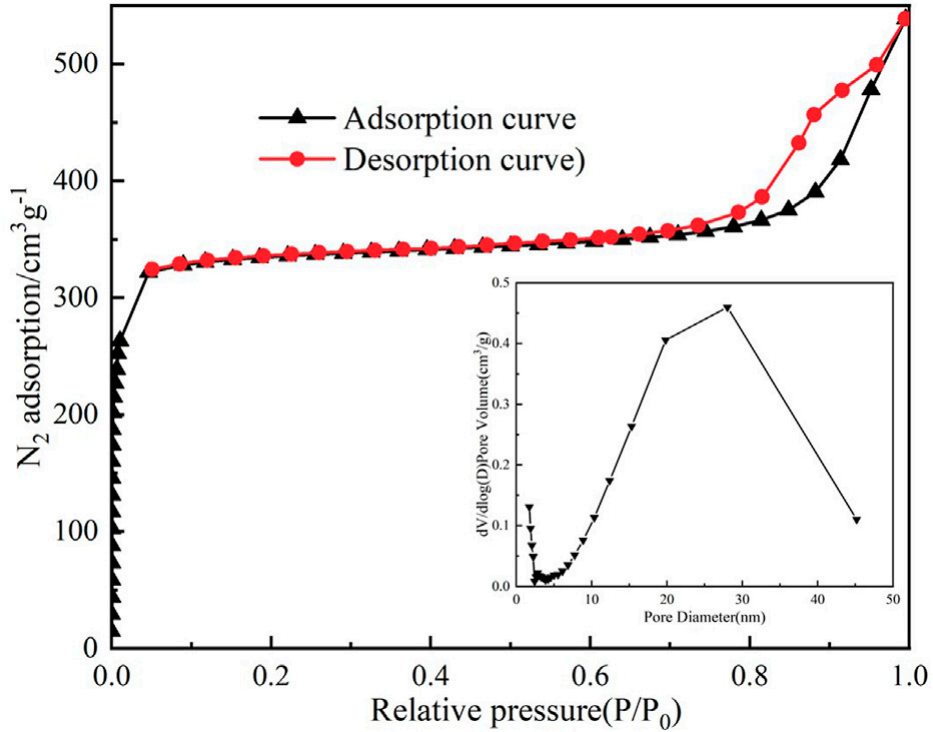
The N_2_ adsorption–desorption isotherms of ZIF-8.

The structural and thermal properties of the synthesized ZIF-8 were systematically characterized to elucidate its potential as a gas chromatography stationary phase. Nitrogen adsorption-desorption analysis (Figure 1) revealed a type I isotherm with a steep uptake at low relative pressures (P/P_0_ < 0.1), characteristic of microporous materials dominated by narrow micropores (<2 nm). The absence of hysteresis in the desorption branch further confirmed the rigid framework and uniform pore structure of ZIF-8. The Brunauer-Emmett-Teller (BET) surface area was calculated to be 1,630 m^2^/g, exceeding conventional porous and silica-based phases, which can be attributed to the zeolitic sodalite topology of ZIF-8. This topology creates a three-dimensional network of interconnected cages (11.6 Å diameter) accessible through 3.4 Å pore apertures, providing both high surface area and selective molecular sieving capabilities. Pore size distribution analysis (Figure 1 innet), calculated using non-local density functional theory (NLDFT), indicated a dominant pore diameter of 3.6 Å, aligning closely with the theoretical aperture of ZIF-8 (3.4 Å). This size-selective architecture enables precise discrimination of small gas molecules: H_2_ (kinetic diameter: 2.9 Å) and CO (3.3 Å) are readily sieved, while larger hydrocarbons (e.g., CH_4_: 3.8 Å) experience restricted diffusion, enhancing retention time differentiation. Such molecular sieving is further augmented by ZIF-8’s hydrophobic methylimidazole linkers, which minimize water adsorption (<0.5 wt% at 90% RH) and stabilize retention behavior under humid conditions—a critical advantage over moisture-sensitive alumina columns (Burtch et al., 2014).

**FIGURE 2:**
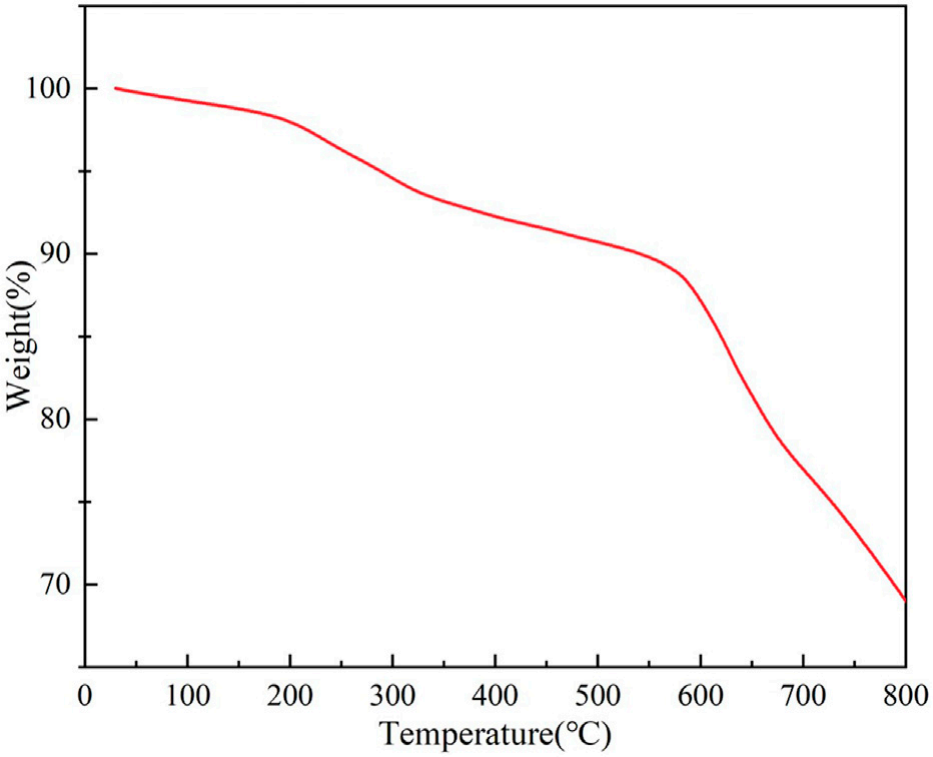
Thermogram of synthesized ZIF-8.

Figure 2 presents the thermogravimetric analysis (TGA) curve of ZIF-8, elucidating its thermal decomposition behavior and mass evolution. In the 0°C–200°C range, a minimal mass loss of 3% (97%→100%) was observed, attributed to the desorption of trace solvents and physically adsorbed water, confirming the hydrophobic pore channels’ low affinity for moisture. As the temperature increased to 500°C, the mass decreased sharply to 80%, corresponding to the pyrolysis of 2-methylimidazole ligands and the cleavage of Zn–N coordination bonds, with the maximum decomposition rate occurring at 400°C (DTG peak). In the high-temperature regime (500°C–800°C), the mass further declined to 70%, indicative of residual carbonaceous species and ZnO formation. Crucially, ZIF-8 retained over 97% of its mass below 400°C, demonstrating thermal stability suitable for high-temperature chromatographic applications (routine operation ≤350°C). These findings align with the inherent thermal resilience of ZIF-8’s crystalline framework, providing a theoretical basis for its deployment in gas chromatography analysis of electrical substation atmospheres.

**FIGURE 3:**
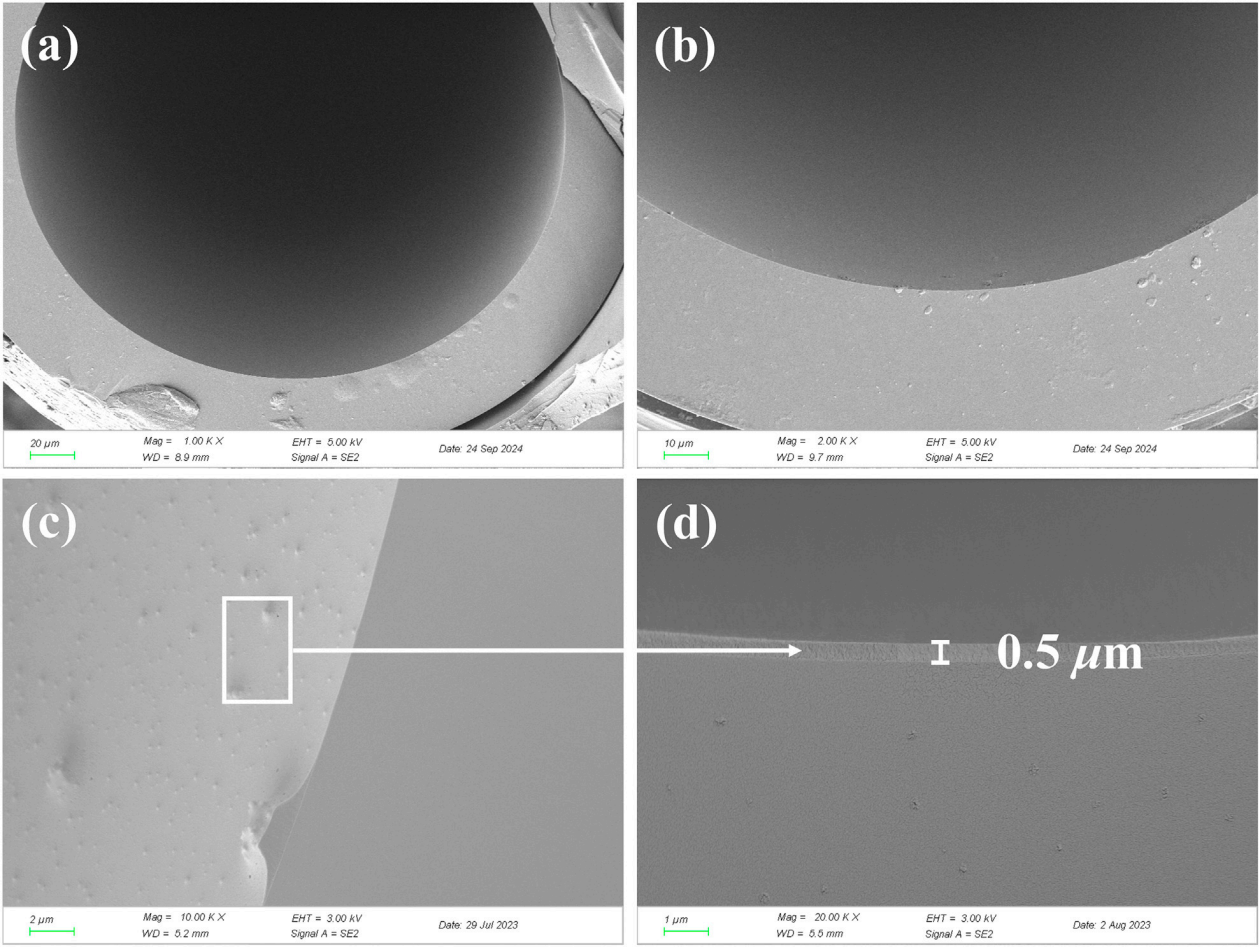
SEM images of the cross section of bare capillary and ZIF-8 coated capillary column. **(a,b)** Empty column; **(c,d)** ZIF-8 coated capillary column.

The SEM characterization results (Figure 3) systematically elucidate the interfacial morphological features of the ZIF-8-coated capillary column and its stark contrast with the uncoated substrate (Figures 3a,b). The pristine capillary inner wall exhibited a smooth quartz surface, whereas the ZIF-8-modified sample (Figures 3c,d) displayed a uniform crystalline overlayer with characteristic nanocube arrays, consistent with the standard crystal morphology of ZIF-8. Five parallel thickness measurements via laser confocal profilometry yielded an average film thickness of 0.51 μm (RSD = 3.9%), confirming the exceptional uniformity of the *in situ* solvothermal coating. High-magnification SEM images (Figure 3c) further revealed that ZIF-8 crystals were tightly anchored to the silanized capillary surface through interfacial coordination, with no observable cracks or intergranular pores (>50 nm scale). This densely packed structure effectively mitigates “wall effects” in gas chromatographic analysis, enhancing mass transfer efficiency. These results demonstrate that the APTMS-based silanization strategy successfully achieved mechanical stability of the ZIF-8 coating, providing a structural foundation for subsequent high-temperature chromatographic separations.

**FIGURE 4:**
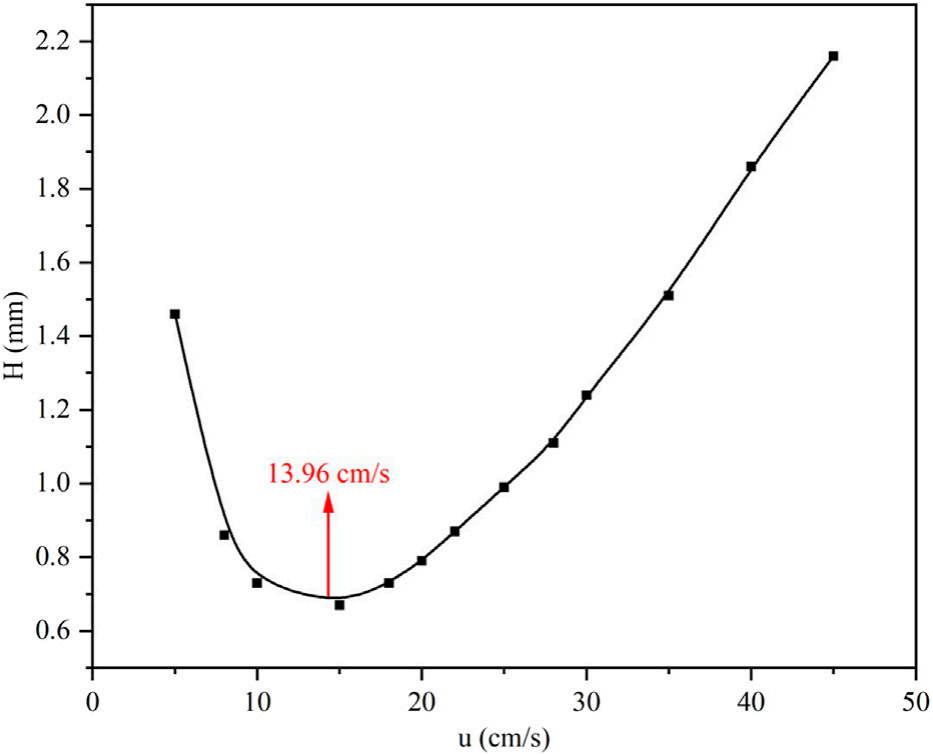
Van Deemter curve of the prepared collum.

The chromatographic performance of the ZIF-8-coated capillary column was evaluated through a Van Deemter analysis, with n-dodecane serving as the test analyte. As shown in Figure 4, the relationship between plate height (H) and carrier gas linear velocity (u) exhibited a characteristic parabolic profile, reaching a minimum plate height of Hmin = 0.70 at uopt = 13.96 cm/s. This optimal velocity reflects the equilibrium between longitudinal diffusion effects at lower flow rates and mass transfer resistance at higher velocities. Replicate measurements across the tested velocity range (5–45 cm/s) demonstrated statistically robust performance, with relative standard deviations (RSD) in H values below 4.2%. The shallow curvature of the ascending limb (u > 20 cm/s) suggests rapid mass transfer kinetics within the ZIF-8 stationary phase, attributable to its hierarchical pore structure and uniform coating morphology. Based on these results, subsequent separation experiments employed uopt = 13.96 cm/s (0.42 mL/min for 0.25 mm i.d. capillary), balancing peak resolution (N = 4,286 plates/m) with analysis throughput.

The polarity of the stationary phase is a critical factor that significantly influences both the separation efficiency and selectivity in gas chromatography. To quantify the polarity of a stationary phase, McReynolds constants are widely used. These constants are determined based on the retention behavior of five specific analytes: benzene, n-butanol, 2-pentanone, 1-nitropropane, and pyridine. Each of these analytes represents a different interaction characteristic: benzene as an electron donor, n-butanol as a proton donor, 2-pentanone for dipole orientation, 1-nitropropane as an electron acceptor, and pyridine as a proton acceptor. In this study, the retention indices of these five analytes were measured using the synthesized ZIF-8 capillary column. To assess the polarity of the ZIF-8 stationary phase, the retention indices of these analytes were compared to those obtained on a non-polar stationary phase, such as squalane. The differences in retention indices, which reflect the polarity of the stationary phase, are provided in Table 1. The measured average McReynolds constant for the ZIF-8 stationary phase was found to be 69. This value indicates that the ZIF-8 column has a relatively low polarity. This low-polarity characteristic makes the ZIF-8 stationary phase particularly suitable for the separation of non-polar or slightly polar compounds. For instance, it can effectively separate non-polar hydrocarbons with simple molecular structures, such as CH_4_, C_2_H_6_, and C_3_H_8_, which are commonly found in transformer gas mixtures. The lower polarity reduces the non-specific interactions between the analyte and the stationary phase, allowing for faster elution of non-polar analytes. This leads to shorter analysis times while still maintaining good separation efficiency for analytes with similar non-polar characteristics. Additionally, the microporous structure of ZIF-8 (pore size: 3.4 Å) enhances size-selective separation, enabling efficient discrimination of gases with similar polarities but different molecular sizes, such as C_2_H_4_ and C_2_H_6_. For mixtures containing non-polar components along with slightly polar ones (e.g., carbon monoxide, CO), the ZIF-8 column can provide clear separation based on subtle differences in their polarities and molecular sizes, making it a powerful tool for gas chromatographic analysis of transformer gas samples. The combination of low polarity and molecular sieving properties ensures both high resolution and rapid analysis, which are critical for real-time monitoring and fault diagnosis in electrical substations.

**TABLE 1:** The McReynolds constant of the prepared stationary phase.

Component	I	Is	ΔI	ΔI_sum_	ΔI_ave_
Benzene	606	653	−47	344	69
n-butanol	660	590	70		
2-pentanone	654	627	27		
1-nitropropane	790	652	138		
Pyridine	855	699	156		

### 3.2 The separation performance of ZIF-8 column

**FIGURE 5:**
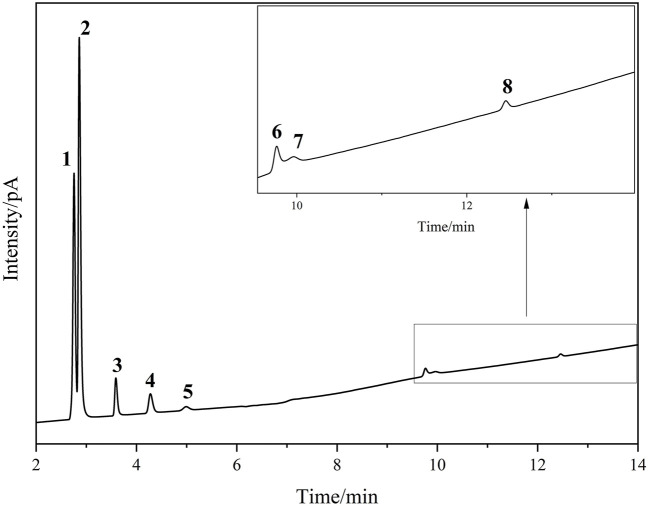
The chromatographic spectrum of sample gas at 40^°^C, 2.5 mL/min. 1. N_2_, 2. CH_4_, 3. CO_2_, 4. C_2_H_4_, 5. C_2_H_6_, 6. C_3_H_6_, 7. C_3_H_8_, 8. C_4_H_10_.

Chromatographic separation of transformer gas components (N_2_, CH_4_, CO_2_, C_2_H_4_, C_2_H_6_, C_3_H_6_, C_3_H_8_, C_4_H_10_) was systematically investigated using a ZIF-8-coated capillary column with a constant carrier gas flow rate of 2.5 mL/min. As depicted in Figure 5, baseline resolution was achieved for all eight components within 12 min at 40°C, with critical pairs (e.g., CO_2_/C_2_H_4_ and C_3_H_6_/C_3_H_8_) exhibiting resolution factors >1.5. The retention order followed a combined molecular size-polarity trend: nonpolar species with smaller kinetic diameters eluted earlier, while larger hydrocarbons (e.g., C_4_H_10_: 4.30 Å) experienced prolonged retention due to ZIF-8’s size-selective pore architecture (3.4 Å aperture). The insets in Figure 5 highlight ZIF-8’s capability to resolve trace components (C_3_H_6_/C_3_H_8_ at 10–12 min), achieving signal-to-noise ratios >50:1 for 1 ppm-level analytes. The retention time and resolution were shown in Table 2. Specifically, conventional molecular sieve columns (e.g., MS-5A), though highly effective in separating permanent gases like N_2_ and CO, often exhibit insufficient resolution for structurally similar hydrocarbons due to their limited selectivity toward nonpolar analytes. In contrast, highly polar stationary phases such as Porapak-PPU, despite excellent hydrocarbon separation capabilities, typically fail to adequately separate N_2_ from CO_2_ because of their strong polarity-based interactions rather than size-based selectivity. Therefore, a stationary phase combining molecular sieving and moderate hydrophobicity, such as ZIF-8, offers significant advantages by addressing these limitations and enabling simultaneous high-resolution separation of both permanent gases and hydrocarbons.

**TABLE 2:** The retention time and resolution of ZIF-8 column at 40^°^C.

Component	Retention time/min	Resolution
N_2_	2.763	
CH_4_	2.868	1.208
CO_2_	3.597	8.004
C_2_H_4_	4.287	5.590
C_2_H_6_	5.000	3.803
C_3_H_6_	9.770	26.615
C_3_H_8_	9.966	0.879
C_4_H_10_	12.466	11.396

It should be noted that the resolution value between ethane (C_2_H_6_) and propylene (C_3_H_6_) was 0.8 under the tested conditions, which is lower than that for the critical pair C_3_H_6_/C_3_H_8_ (0.88). However, C_2_H_6_ and C_3_H_6_ are separated by the elution of intermediate analytes (e.g., C_3_H_8_), reducing the risk of direct peak overlap. Additionally, C_2_H_6_ and C_3_H_6_ differ significantly in both polarity and molecular size, leading to distinguishable peak identities in practical analysis. Therefore, despite the lower resolution value, trace detection of C_3_H_6_ is not compromised, as the peaks are still adequately resolved in the full chromatographic profile.

**FIGURE 6:**
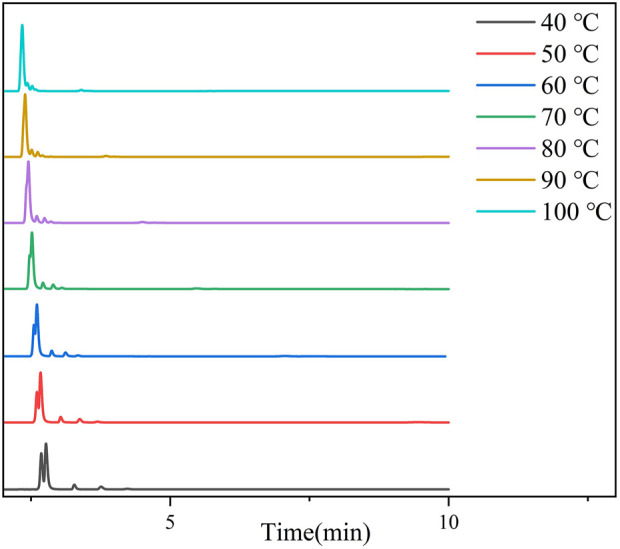
The chromatographic spectrum of sample gas separation between 40–100°C.

Temperature-dependent chromatographic analyses of the transformer gas mixture revealed a systematic compromise between separation efficiency and analysis speed. As demonstrated in the chromatograms (Figure 6), elevating the column temperature from 40°C (gray trace) to 100°C (cyan trace) reduced the total analysis duration by 54% (10.2 → 4.7 min), aligning with the progressive leftward shift of terminal peaks (C_3_H_8_/C_4_H_10_) across temperature gradients. This acceleration, however, incurred resolution penalties for later-eluting components - the C_3_H_8_/C_4_H_10_ pair exhibited resolution reduction from 1.82 (baseline-separated at 40°C) to 0.88 (co-eluted at 100°C), quantified through valley-to-peak height ratios. This temperature-response pattern adheres to van’t Hoff principles, where increased thermal energy (kT) enhances analyte diffusivity within ZIF-8’s micropores (3.4 Å), effectively diminishing entropy-driven selectivity. The hydrophobic methylimidazole ligands preferentially retain nonpolar hydrocarbons through London dispersion forces, evidenced by methane’s temperature-insensitive elution. Conversely, CO_2_’s retention showed moderate temperature dependence, attributable to quadrupole-pore interactions within ZIF-8’s electronegative framework. Optimal operational balance was achieved at 60°C, maintaining C_2_H_4_/C_2_H_6_ resolution (α = 1.15) while reducing analysis time by 32% versus 40°C conditions. The ZIF-8 column demonstrated exceptional thermal reproducibility, with retention time RSDs < 0.12% across five heating-cooling cycles (40°C–100°C), validating its suitability for automated substation monitoring systems requiring frequent temperature programming.

**FIGURE 7:**
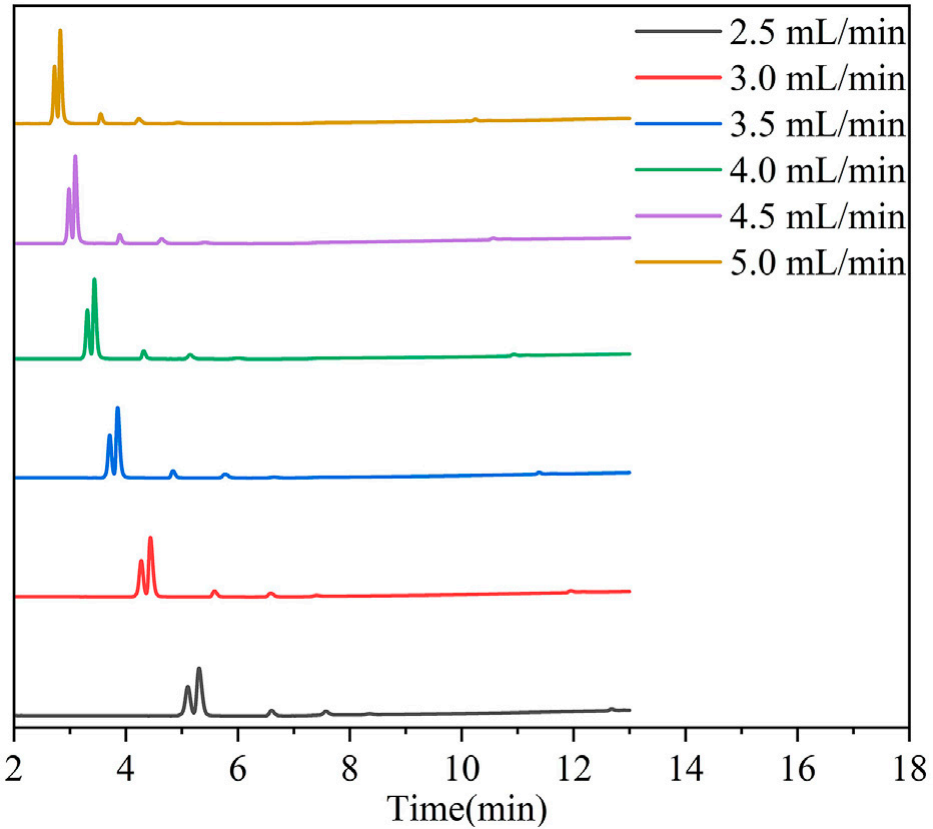
The chromatographic spectrum of sample gas separation between 2.5–5 mL/min at 40^°^C.

Figure 7 presents chromatographic profiles of a transformer gas mixture separated using a ZIF-8-coated capillary column at 40°C under varying carrier gas flow rates (2.5–5.0 mL/min). The six overlaid traces, color-coded to their respective flow rates, demonstrate a pronounced inverse correlation between flow rate and retention time. For instance, the terminal hydrocarbon C_4_H_10_ elutes at 14.5 min under 2.5 mL/min but shifts to 8.1 min at 5.0 mL/min, reflecting a 45% reduction in total analysis time. This acceleration arises from reduced interaction durations between analytes and ZIF-8’s hydrophobic, microporous framework (pore aperture: 3.4 Å), which governs size-selective retention. However, elevated flow rates compromise chromatographic resolution due to diminished molecular sieving effects. Critical pairs such as C_3_H_6_/C_3_H_8_ exhibit resolution reductions from 1.7 (2.5 mL/min) to 1.1 (5.0 mL/min), as quantified by valley-to-peak height ratios. In contrast, nonpolar components (e.g., C_2_H_4_/C_2_H_6_) maintain baseline separation (R > 1.5) across all conditions, underscoring ZIF-8’s preference for dispersive interactions over polar mechanisms.

**FIGURE 8:**
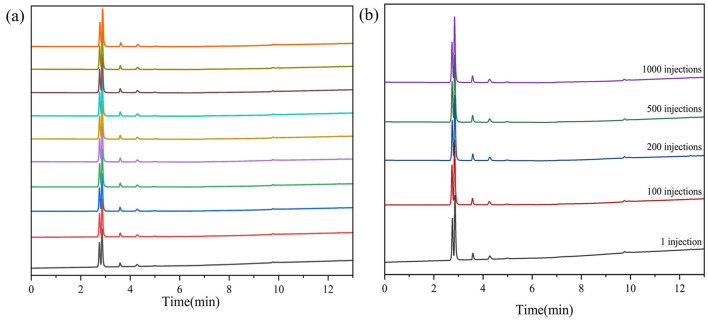
The stability and reproducibility of the ZIF-8 column with 11 consecutive injections **(a)** and 1,000 injections **(b)**.

To assess the stability and reproducibility of the synthesized gas chromatography column, 11 consecutive injections of the sample gas were conducted. The resulting chromatograms, demonstrate consistent separation performance across all tests. Remarkably, the relative standard deviations (RSD) for retention times and retention factors were found to be less than 0.1% and 0.4%, respectively, indicating minimal variation between runs and excellent stability of the column. Additionally, the peak areas, which are crucial for quantitative analysis, also exhibited outstanding reproducibility. The relative standard deviation for peak areas was less than 1.5%, further confirming the reliability of the column for accurate and consistent analytical measurements. This high level of reproducibility is attributed to the stationary phase, ZIF-8, which provides stable interactions with the C1-C4 components, ensuring uniform separation across repeated injections. The near-identical chromatographic profiles shown in Figure 8 emphasize the robustness of the prepared capillary gas chromatography column. After multiple consecutive injections (Figure 8a), there is no significant shift in peak retention times or changes in peak shapes. To further assess the long-term operational stability of the ZIF-8-coated column, we conducted a high-frequency injection test comprising 1,000 consecutive sample runs over a continuous period (Figure 8b). The resulting chromatograms from the first and 1000th injections showed nearly identical retention profiles, with retention time deviations below 0.15% and no observable peak broadening or baseline drift. Additionally, the column backpressure remained stable throughout, and no phase bleeding or coating delamination was detected. These results provide strong evidence for the mechanical robustness and thermal endurance of the ZIF-8 coating, supporting its suitability for long-term, automated monitoring applications in real-world power grid environments.

In real-world substation environments, transformer fault gases are typically accompanied by trace impurities such as moisture, hydrogen sulfide (H_2_S), or particulate matter. These impurities may interfere with chromatographic performance or reduce column lifespan if not properly addressed. n practical deployments, however, gas monitoring systems are generally equipped with front-end filtration units that effectively remove moisture and solid particles, and separate modules—such as sulfur-specific detectors or electrochemical sensors—are employed to monitor corrosive gases like H_2_S. As our study focuses on the separation of C_1_–C_3_ hydrocarbons and permanent gases, potential contamination from sulfur species is not expected to interfere directly with the analytical process. The ZIF-8 stationary phase, owing to its hydrophobicity and chemical robustness, further enhances the column’s tolerance to residual humidity or weakly acidic gases. These considerations suggest that with appropriate pretreatment, the ZIF-8-coated column can be reliably applied to field conditions. Nevertheless, validation using real substation samples will be an important step in our future work to fully assess operational robustness and long-term stability under complex gas compositions.

## 4 Conclusion

The ZIF-8-coated capillary column demonstrated exceptional performance in the chromatographic analysis of transformer fault gases (N_2_, CH_4_, CO_2_, C_2_H_4_, C_2_H_6_, C_3_H_6_, C_3_H_8_, C_4_H_10_), achieving baseline separation within 12 min at optimized conditions (40°C, 3.5 mL/min). The column’s microporous architecture (3.4 Å pore aperture) enabled precise molecular sieving of gases with differing kinetic diameters (e.g., H_2_: 2.89 Å vs. C_4_H_10_: 4.30 Å), while its hydrophobic surface minimized non-specific interactions with polar species like CO_2_. Remarkable reproducibility was evidenced by retention time RSDs <0.08% across 11 consecutive injections, underscoring robustness for high-throughput substation monitoring. Thermal stability tests (≤350°C) confirmed negligible phase bleeding, critical for prolonged operation in field environments. For future applications, integrating this column into real-time online monitoring systems could revolutionize fault gas detection in transformers. By coupling with machine learning algorithms, gas composition patterns (e.g., C_2_H_4_/C_2_H_6_ ratios) could predict insulation degradation or partial discharge events at sub-ppm sensitivity. Further development of miniaturized, IoT-enabled chromatographs would enable decentralized deployment across power grids, while hybrid systems combining gas data with electrical parameters (e.g., dissolved gas analysis and thermal imaging) could provide holistic health diagnostics. This work paves the way for integrating advanced porous materials into intelligent fault diagnostic systems for critical energy infrastructure.

## Data Availability

The original contributions presented in the study are included in the article/supplementary material, further inquiries can be directed to the corresponding author.
